# Concentration of potentially toxic elements in soils of an intensively agricultural and industrial watershed

**DOI:** 10.1007/s10653-026-03400-8

**Published:** 2026-08-07

**Authors:** Luís Reynaldo Ferracciú Alleoni, Evandro Barbosa da Silva, Adriele Laena Ferreira de Moraes

**Affiliations:** 1 Luiz de Queiroz College of Agriculture, University of São Paulo, Piracicaba, 13418900 Brazil; 2 Mack Technologies, Melbourne, FL 32904 USA

**Keywords:** Potentially toxic elements, Tropical soils, Environmental risk assessment, Environmental monitoring

## Abstract

Soil contamination by potentially toxic elements (PTEs) can alter the structure and functioning of ecosystems and pose risks to public health. In this context, micro.watersheds are important units of analysis, as they integrate natural and anthropogenic processes that influence the distribution of these elements in the environment. In general, environmental agencies establish soil quality guidelines valid for large territorial areas, such as states or countries.However, studies at smaller scales are still limited, especially in micro–watersheds. Our objectives were to quantify the concentrations of arsenic, cadmium, cobalt, copper, chromium, lead, nickel, and zinc in the soils of the Guamium river watershed, located in a Brazilian region with metalwork, boiler making and foundry industries, steel mills, sugarcane and ethanol plants, and the automotive sector; to compare these concentrations with the guidelines established by the Environmental Company of the State of São Paulo; and to establish quality reference values for PTEs in the micro–watershed. Arsenic and cooper presented the highest concentrations, with values equal to or greater than the prevention value established by the environmental agency. The study contributes to the understanding of the natural and anthropogenic variability of PTEs in tropical soils and provides support for environmental monitoring, risk assessment, and the definition of more representative reference values on a regional scale.

**Supplementary Information:**

The online version contains supplementary material available at 10.1007/s10653-026-03400-8.

## Introduction

Potentially toxic elements (PTEs) are natural constituents of rocks and sediments and their concentrations in soils depend on the parent material, formation processes and the proportion and composition of the solid phase (Wang et al, 2023). Anthropogenic activities, such as industrial emissions, urban effluent discharge, and the use of pesticides, can increase PTE concentrations in the soil (Monaci & Baroni, 2025). In this context, the assessment of PTE levels in the soil is an useful tool in environmental studies related to contamination and pollution (Fernandes et al., 2018; Pereira et al., 2020), especially for establishing guideline values in the soil (Fernandes et al., 2018).

Soil contamination by PTEs is a global concern, and the impact of these contaminants on the soil depends on their quantity, chemical form, and availability in the soil. High concentrations of PTEs can pose risks to the environment and human health (Dias et al., 2022; Teixeira et al., 2021), and the increasing replacement of native vegetation by agricultural, residential, and industrial areas may intensify soil contamination by PTEs (Yaşar Korkanç et al., 2024) 

In general, the concentration of PTEs increases in regions with greater human occupation, and these elements are usually retained in the uppermost layers even at high levels, without reaching the entire soil profile. However, the presence of high concentrations of PTEs weakly bound to soil colloids can result in serious risks of leaching along the profile and migration into groundwater (Liu et al., 2024). Thus, determining the levels of these elements in watersheds is important for assessing the environmental impact and supporting the development of technologies aimed at protecting soil and water resources.

The Piracicaba Metropolitan Region (PMR), in the state of São Paulo, Brazil, comprises 24 municipalities, has an estimated population of 1.5 million inhabitants, an annual GDP of approximately US$ 15 billion (PMP, 2024), and is home to a diversified industrial park, which includes metalwork, boiler making and foundry industries, steel mills, sugar and ethanol plants, and the automotive sector, as well as markets and other establishments. The combination of these agricultural, urban, and industrial activities constitutes potential sources of contamination by PTEs in the soil and water resources.

Environmental agencies establish guiding values for soil quality, including the reference value (QRV), which corresponds to natural concentrations in areas without anthropogenic influence. The QRVs of natural inorganic substances in soils are defined from the statistical interpretation of samples collected from the main soil types in the study area, in the 0–0.2 m layer (CONAMA, 2009), and are determined based either on the 75th (upper quartile) or 90th percentiles, using the EPA 3051 method (USEPA, 2007).

In addition to the QRV, the prevention value (PV) corresponds to the limit concentration of a substance in the soil capable of preserving its main functions, and represents an alert level. On the other hand, the intervention value (IV) is the concentration of a substance in the soil or groundwater above which there are potential direct and indirect risks to human health, with specific values for agricultural, residential, and industrial areas (CONAMA, 2009).

The adoption of a single QRV for extensive areas does not adequately represent the geochemical diversity of soils, while regionalized QRVs are more effective for environmental monitoring and for distinguishing between natural and anthropogenic concentrations of PTEs in soils (Cardoso et al., 2023). In this context, the watershed basin constitutes an appropriate spatial unit for defining QRVs.

Our objectives in this study were to: (i) quantify the concentrations of arsenic (As), cadmium (Cd), cobalt (Co), copper (Cu), chromium (Cr), lead (Pb), nickel (Ni), and zinc (Zn) in the Guamium river watershed, a tributary of the Piracicaba River; (ii) compare the concentrations with the values established by the São Paulo State Environmental Agency; and (iii) establish reference values for PTEs in the micro–watershed.

The results of the study can contribute to environmental monitoring and sustainable soil management. Our hypothesis is that PTE concentrations in the watershed increase as areas previously occupied by native vegetation are replaced by agricultural, residential, and industrial areas.

## Materials and methods

### Study site and sampling

**Fig 1:**
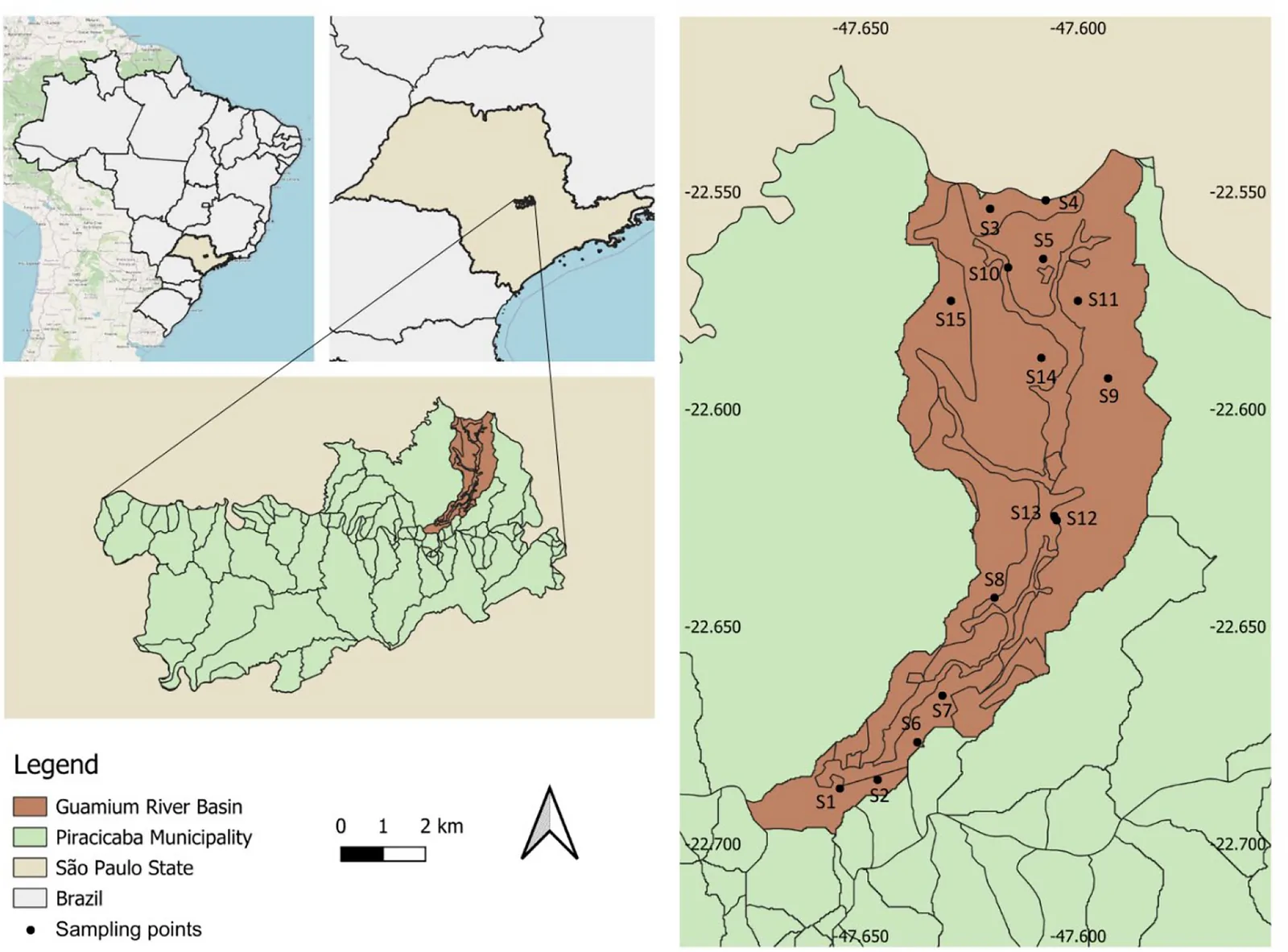
**-** Location and soil sampling points of the Guamium watershed microbasin, Piracicaba, SP, Brazil

The Guamium River basin is located entirely within the municipality of Piracicaba, São Paulo state, Brazil, between longitudes 47º34'20.85" W–47º40'29.68" W and latitudes 22º32'18.64" S–22º41'43.14" S, and covers ​​7051 ha, of which 481 ha are located within an urban area (PMP, 2024) (Fig. 1). According to the Köppen classification, the region's climate is Cwa (tropical highland) (Alvares et al., 2013), with an average temperature of 22.5 ºC and an annual rainfall of 1300 mm, concentrated mainly between the months of October and March.

The basin exhibits diverse land use, with a predominance of agricultural areas dedicated to sugarcane (*Saccharum officinarum*) cultivation, occupying approximately 45% of the territory. In contrast, the urban area corresponds to only 6% of the total area. To characterize the watershed's soils, samples were collected at 15 representative points, encompassing the main soil classes identified in the region (Fig. 1). Samples were collected along the basin at depths of 0–0.1 m; 0.1–0.2 m; 0.2–0.3 m; 0.3–0.4 m; 0.4–0.6 m; 0.6–0.9 m; and 0.9–1.2 m (Fig. 1). At each point, six subsamples were obtained to form a composite sample. The predominant soil classes were Oxisol and Entisol.

### Soil chemical characterization

Soil samples were air–dried and then passed through a 2 mm mesh sieve. Values of pH and contents of organic carbon (OC), phosphorus (P), potassium (K), calcium (Ca), magnesium (Mg), aluminum (Al), H + Al, copper (Cu), iron (Fe), manganese (Mn), and zinc (Zn) were then determined (Raij et al., 2001). The results of the exchange complex were used to calculate the cation exchange capacity (CEC). Contents of Al, Fe, and Mn were extracted by sulfuric acid, while Si contents were extracted by sodium hydroxide according to Teixeira (2017), and the weathering indices Ki = 1.7 × SiO_2_/Al_2_O_3_ and Kr = 1.7xSiO_2_/(Al_2_O_3_ + 0.64 Fe_2_O_3_) were calculated according to Vettori (1969). Crystalline oxide contents were extracted with sodium dithionite–citrate–bicarbonate (Loeppert & Inskeep, 1996), while amorphous oxide contents were extracted with ammonium oxalate and oxalic acid and determined by the method described by Bertsch and Bloom (1996) and Loeppert and Inskeep (1996).

The pseudototal concentrations of As, Cd, Co, Cu, Cr, Pb, Ni, and Zn were extracted according to the EPA 3051A method by microwave digestion (USEPA, 2007). The As content was determined by atomic fluorescence spectrometry (AFS), and atomic absorption spectrometry (AAS). Graphite furnace coupled to AAS (GF-AAS) (Perkin-Elmer, Shelton, USA) determined the contents of the other elements when their concentrations were below the AAS detection limit. For quality control, certified soil samples were included (Soil Montana, NIST SEM 2711).

Prior to analysis, the GF-AAS instrument was calibrated using multi–element standard solutions within appropriate concentration ranges. Calibration curves were constructed using at least five concentration levels. Instrument accuracy and analytical precision were verified throughout the analytical sequence using certified reference materials and procedural blanks. Limit of quantification was determined as the lowest concentration of the calibration solutions, which was 1 mg L^−1^. All reagents were purchased from Synth (Diadema, São Paulo, Brazil), including nitric acid (HNO_3_, 65.0%, PA-ACS grade) and hydrochloric acid (HCl, 38.0%, PA-ACS grade).

The CONAMA (2009) methodology was used to obtain a value that represents the entire watershed. Reference values for inorganic substances in the soil are established from the statistical interpretation of analytical results from representative samples at a depth of 0 to 0.2 m (CONAMA, 2009). These values are obtained from the 75th (upper quartile) and 90th percentiles, and concentrations are extracted using the EPA 3051A method. Outliers identified by the statistical method were excluded from the data matrix to obtain a more realistic water quality condition of the Guamium watershed and the subsequent comparison with the background concentration of São Paulo.

## Results and discussion

### Soil characterization of the guamium watershed

The pH of most soil samples was low, according to the classification of Alvarez et al. (1999) (Table 1). Similar values were obtained for soils from other Brazilian states such as Mato Grosso and Rondônia (Santos & Alleoni, 2013), Espírito Santo (Paye et al., 2010), Pará (Fernandes et al., 2018) and in the Verruga River basin in the state of Bahia (Cardoso et al., 2023). Under low pH conditions, many PTEs are in more soluble forms (Jalali & Najafi, 2018), which can increase their mobility and bioavailability in the soil. Clayey texture was predominant in relation to sand and silt fractions, but some soils presented high sand contents, and the predominant textural class was clayey-sandy. The total CEC (pH 7) had an average value of 66 mmol_c_ kg^−1^.

**Table 1:** Average natural levels of potentially toxic elements in the most superficial soil layer (0–0.2 m) from the Guamium watershed compared with data compiled from international bibliographic references

Location	As	Cd	Co	Cr	Cu	Ni	Pb	Zn
	mg kg^−1^							
Guamium	7.7	< LQ^(11)^	4.4	30.2	33.2	9.84	16.8	33.4
Australia^(1)^	20	1	–	100	100	60	150	1400
China^(1)^	9.2	0.1	–	53.9	20.0	23.4	23.6	67.7
Ireland^(2)^	5.2	0.5	–	49.5	16.9	13.5	30.4	70.3
USA^(1)^	0.1–93	1.6	20	37	17	13	16	48
SP^(3)^	2.75	0.5	15.7	22.7	53.3	13.0	11.1	45.6
MG^(4)^	3.8 – 50.6	0.5	16.5	100.1	30.9	30.1	3.9	13.1
PR^(5)^	-^(10)^	0.1	23.8	105.0	141.7	34.1	23.2	75.2
ES^(6)^	6.8	< LD^(12)^	8.6	41.0	5.5	6.6	8.8	22.6
PE^(7)^	0.44	0.62	3.5	27.1	7.2	6.0	10.7	22.5
PA^(8)^	0.7	0.2	–	14.7	5.2	0.6	2.8	4.5
RO e MT^(9)^	–	< LD	20	39	17	1.3	8.1	6.8

^(1)^Chen et al.(1991) and Guilherme et al. (2005), ^(2)^Salonen et al. (2007), ^(3)^CETESB (2001), ^(4)^Caires (2009), ^(5)^MINEROPAR (2005), ^(6)^Paye (2008), ^(7)^Biondi (2010), ^(8)^Fernandes et al. (2018), ^(9)^Santos & Alleoni (2012), ^(10)^Data not evaluated. ^(11)^Below the limit of quantification, ^(12)^Below the limit of detection. São Paulo (SP), Minas Gerais (MG), Paraná (PR), Espírito Santo (ES), Pernambuco (PE), Pará (PA), Rondônia (RO), Mato Grosso (MT) are Brazilian states

Organic carbon (OC) levels were higher than the average values for soils from humid tropical regions. Both silicate minerals in the clay fraction and OC influence the soil's capacity to retain and complex cationic EPTs, due to the greater number of reactive surfaces and available functional groups (Palansooriya et al., 2020). The average values of the weathering indices Ki and Kr were 0.9 and 0.2, respectively, indicating highly weathered soils (Resende; Santana, 1998). The average levels of crystalline oxides of Al, Fe, and Mn were 16, 81, and 0.6 g kg⁻^1^, while the levels of amorphous oxides were 5.3, 56, and 1.5 for Al, Fe, and Mn, respectively. The Fe content averaged 432 g kg⁻^1^, with most soils classified as rich in iron (Embrapa, 2025).

### Concentrations of PTEs

Most PTE concentrations in the soils of the Guamium watershed had values lower than the background concentrations established for the state of São Paulo (CETESB, 2021), except for As and Cu (Table 2). PTEs from natural sources and anthropogenic activities can accumulate in soils and impact environmental quality and human health (Parida & Patel, 2023; Sharafi & Salehi, 2025).

**Table 2:** Levels of potentially toxic elements in the soils of the Guamium Watershed, Piracicaba, SP, Brazil

	As	Cd	Co	Cr	Cu	Ni	Pb	Zn
	mg kg^−1^							
Average	6.9	< LQP	3.9	28.8	28.4	6.5	5.4	27.6
Minimum	1.5	< LQP	0.4	15.9	12.2	0.17	3.2	13.2
Maximum	18.3	< LQP	14.4	51.7	94.9	34.8	32.0	79.4
P 75	9.3 (3.5)	0.5^1^ (< 0,5)	6.1 (13)	35.9 (40)	40.4 (35)	11.4 (13)	21.9 (17)	47.2 (60)
P 90	14.8	0.5	6.9	38.2	50.9	27.1	31.8	64.7

^1^Practical quantification limit; Values in parentheses to the 75th percentile (P75) established by CETESB (2005)

In general, the average concentrations of PTEs were close to the national and international averages (Table 2), indicating that the basin does not exhibit altered levels for most of the elements evaluated. Lower concentrations of PTEs, except for Cd, Co, and Cu, were found in soils from Mato Grosso and Rondônia (Table 2) (Santos & Alleoni, 2013). This pattern reinforces that certain elements may exhibit regional enrichment associated with specific lithological and anthropogenic characteristics, which may also explain the observed elevations for As and Cu in the Guamium basin.

The high concentrations of As and the lack of correlation with depth (Table 1) indicate that the parent material possibly had high concentrations of the element. Parent material is one of the main components in the distribution of As (Wang et al., 2023). In this context, the high values ​​in samples located near the Guamium stream may be associated with As precipitation. Furthermore, potential anthropogenic sources include historical use of As–based pesticides, atmospheric deposition from industrial activities, and the burning of fossil fuels (Teixeira et al., 2020).

Natural or anthropogenic emissions caused by large–scale phenomena, such as floods, erosion, landslides, and water scarcity, affect the mobilization and immobilization balances of As in the environment (Teixeira et al., 2020). Arsenic can flow into rivers, percolate into groundwater, or be absorbed by plants, allowing it to enter the food chain and posing serious risks to human health. Its mutagenic and carcinogenic nature, coupled with the growing number of contaminated areas, makes it one of the main current environmental concerns (Oliveira et al., 2021).

Point S10 (Entisol) stands out as the most critical in our study, as it is located near the stream and, therefore, more subject to interactions between sediment and water. Under low redox potential conditions (reducing environments), the toxic and mobile cationic species As^3+^ predominates. Under these conditions, reductive dissolution of Fe and Al hydroxides can occur and release previously adsorbed As and increase its bioavailability in the environment (Mensah et al., 2022). The presence of As has also been recorded in water samples from other Brazilian rivers and streams, with concentrations exceeding the quality standards established by national and international organizations (Bundschuh et al., 2021; Faria et al., 2023).

Cadmium concentrations were below the detection limit, consistent with its low natural abundance and relatively high mobility in the soil (Kubier et al., 2019). Although the concentrations were low, Cd is highly toxic to plants and other organisms (Soni et al., 2024). In a study of competitive adsorption of Cd, Cu, Ni, and Zn in the surface layer (0–0.2 m) of soils in the state of São Paulo, Cd was the least retained element (Moreira & Alleoni, 2010), reinforcing its high mobility and low affinity for reactive soil components.

Cobalt concentrations were lower than those observed in soils from the state of São Paulo (Table 2). In both soil classes, there was a positive correlation of Co concentrations with the levels of oxides and hydroxides of Al, Fe, and Mn, which highlights the role of these constituents in the geochemical control of the element. Iron and manganese (hydr)oxides favor the retention and distribution of Co in soils and sediments. During the weathering, Co can be incorporated into the mineral structure and replace Mn, which contributes to its stabilization in the soil (Ziwa et al., 2020).

The average Cr concentration was higher than the average for soils in São Paulo (CETESB, 2021), but lower than the state QRV (Table 2). Compared to other Brazilian states, the results were close to those of Espírito Santo (Paye et al., 2010), Pernambuco (Biondi, 2010), Mato Grosso and Rondônia (Santos & Alleoni, 2013), and higher than that of the state of Pará, in the Amazon region (Fernandes et al., 2018). In the soil, Cr occurs mainly as Cr^3+^ and Cr^6+^, with Cr^3+^ being the more stable and predominant form, while the Cr^6+^ species is more mobile, soluble, and toxic (Coyte et al., 2020; Hossini et al., 2022).

The Cr content in the Entisol showed a positive correlation with soil depth (Table 2), indicating possible leaching of the element, which could result in water contamination, since this soil is located near the Guamium stream. The presence of Cr^6+^ in soil and groundwater may be associated with anthropogenic activities such as coal combustion, mining, leather tanning, paint manufacturing, and phosphate fertilizer production (Hausladen et al., 2018; Vasileiou et al., 2019; Wang et al., 2022).

The mobility and geochemical behavior of Cr are primarily controlled by pH and redox conditions. Cr^6+^ occurs in neutral to alkaline environments (pH ≥ 7) and oxidizing conditions, while Cr^3+^ is more stable in acidic and reducing environments (Wang et al., 2022). This behavior occurs in Entisols due to flood cycles and reducing conditions. Cr^6+^ presents a high risk due to its strong oxidizing power, which can cause adverse effects such as asthma, cancer, allergic reactions, neurological and cardiovascular disorders, as well as organ failure (Guo et al., 2021).

Copper presented high concentrations in the Guamium watershed (Tables 1 and 2), with maximum values exceeding the PV established by CETESB (2021). The high concentrations of Cu may be related to the high concentrations of organic matter (OM) and oxides of Fe, Al, and Mn. Cu has an affinity for OM, and its interaction is mediated by the formation of stable complexes with carboxylic and phenolic functional groups of humic substances, which favors its retention in the surface layers (Huang et al., 2018).

In the study area, the high concentrations of Cu may be associated with the prolonged use of organic fertilizers, since fertilizing the soil with manure increases the availability of Cu (Araújo et al., 2019). Guan et al. (2018) reported increased Cu concentrations in Chinese soils subjected to more than 20 years of manure application. However, the absence of variation in Cu concentrations along the profile suggests that the high levels observed are also associated with the parent material.

The Pb levels in the surface layers were similar to those of soils in the state of São Paulo (CETESB, 2021). However, the concentrations exceeded the QRVs at two sampling points (S11 and S12), which may indicate possible soil contamination (CETESB, 2021). Similar results were reported for soils in the states of Mato Grosso and Rondônia (Santos & Alleoni, 2013), Espírito Santo (Paye et al., 2010), and Paraná (MINEROPAR, 2005). On the other hand, Caires (2009) observed that the soils of the state of Minas Gerais presented lower Pb levels, with Pb, along with Cd, being one of the elements with the lowest concentrations in that state.

### Effect of urbanization on EPT concentrations

The correlation between As levels and distance from the Guamium river was negative; so, As concentration decreased as the sampling points approached the urban/industrial area, indicating that agricultural sources were a major contributor to the high As levels. The Guamium river is located in a rural area with small forest fragments and agricultural areas; the closest point to the river was S4, and the furthest was S1, located in an urban area. The distance between the two points was approximately 15 km (Table 2).

Arsenic contamination in the groundwater of the Bengal Basin in India, one of the most severe ever recorded, has a predominantly geogenic origin, although the contribution from anthropogenic sources is also relevant (Chakraborty et al., 2015). In this basin, arsenic is released from the aquifer matrix into the groundwater under favorable geochemical conditions. In China, Luo et al. (2010) pointed to different sources of arsenic contamination in distinct regions. In the main Beijing basin, concentrations were higher than the city's reference levels in soil and in water, and were associated with gold and iron mining activities. In the groundwater of Shanxi province, Li et al. (2021) observed that contamination was related to inadequate land use practices during rapid urbanization and industrialization.

The Guamium basin region is predominantly occupied by sugarcane cultivation areas, with sporadic presence of corn (*Zea mays*). Even with agricultural land use, no correlation was observed between distance and the content of micronutrients and potential contaminants, such as Co, Cu, Ni, and Zn (Table 2). However, points S11 and S12 concentrated the highest levels of these elements in the watershed. The association between Co, Cu, Ni, and Zn may indicate both pedogenetic characteristics and anthropogenic influence; furthermore, high concentrations may also be related to the application of fertilizers and pesticides (Ferreira et al., 2023; Reis et al., 2017).

In the Iguaçu River basin (25º32'16" S, 49º13'32" W), which receives high sewage loads from the metropolitan region of Curitiba, capital of the state of Paraná, Brazil, (Sodré et al., 2012) reported that the urbanization process influenced urban Cu contamination, promoting an increase in the element's content in the water and its accumulation in downstream sediments. In turn, Reis et al. (2017) observed that high concentrations of Cu found in the Matipó River basin, state of Minas Gerais, Brazil (20º20'0" S, 42º15'0" W) were associated with local geological characteristics, but predominant agricultural practices in the region may also contribute to the input of PTEs to water bodies, since fertilizers and pesticides can be transported by surface runoff.

### Comparison between background concentration and the reference value for water quality in the guamium river basin

When all concentrations of a substance are below the practicable quantification limit (PQL), the QRV is defined as equal to the PQL, according to the CONAMA (2009) resolution. Most soils did not vary between the surface and subsurface layers. All samples had Cd concentrations below the quantification limit. Therefore, the PQL was established as the QRV, and Cd was excluded from other statistical interpretation procedures (CONAMA, 2009).

The 90th percentile values were higher than the 75th percentile for all elements. This occurs because the 90th percentile covers a wider range of values than the 75th percentile, thus broadening the concentration intervals. Therefore, concentrations that would be classified as high based on the 75th percentile are considered normal at the 90th percentile. However, high values defined by the 90th percentile do not necessarily indicate anthropogenic contamination, as they may simply reflect the natural variability of the soils (Fernandes et al., 2018).

There were differences between the concentrations of EPTs in the soil of the Guamium watershed and the values established by CETESB (2021). Arsenic and Ni showed the greatest differences, with P90 values close to the prevention value (CETESB, 2021). However, CETESB, the environmental agency of the state of São Paulo, adopted P75, which is a more restrictive criterion, for defining reference values.

Arsenic presented values higher than the background values of CETESB (2021). P75 was approximately three times higher (9.3 mg kg^−1^) than the QRV of São Paulo (3.5 mg kg^−1^), while P90 (14.8 mg kg^−1^) approached the prevention value (15 mg kg^−1^) (Table 1). The concentrations of As in uncontaminated soils can vary between 1 and 20 mg kg^−1^ (Kabata-Pendias, 2010), since local geology, rock weathering, and volcanic activity are natural sources responsible for As enrichment (ATSDR, 2019; Fernandes et al., 2018). Compared to soils from the state of Espírito Santo (Paye et al., 2010), the P90 values are similar, but P75 was higher than the QRV of Guamium. It is worth noting that the reference value for the state of São Paulo is among the most restrictive in the world, surpassed only by some regions of the USA (CETESB, 2021).

The P90 of the QRV for Ni was close to the prevention value established by CETESB (2021), while P75 remained below the background concentration of São Paulo (Table 1). The background concentrations of PTEs soils from the states of Mato Grosso and Rondônia (Santos & Alleoni, 2013), obtained by the EPA 3051 method (HNO_3_:HCl 3:1 v/v), were lower than the values for São Paulo and the Guamium basin. The state of Espírito Santo established a similar value (17.2 mg kg^−1^) (Paye et al., 2010).

Copper P90 was close to the PV, while P75 was higher than the baseline value for the state of São Paulo (Table 1). Lower Cu concentrations were reported for soils in the states of Mato Grosso and Rondônia (Santos & Alleoni, 2013) compared to the values from the Guamium basin and CETESB, when using the EPA 3051 method. On the other hand, digestion in aqua regia resulted in values similar to those of São Paulo, but lower than those found in this study.

The Pb content corresponding to P90 was approximately double the background value for the state of São Paulo (Table 1). The background value established for Espírito Santo (8.9 mg kg^−1^) is lower than that adopted by CETESB (Paye et al., 2010), as are the values reported for Mato Grosso and Rondônia, regardless of the extractor used, and higher than the value for Amazonian soils (Nascimento et al., 2018). In contrast, when compared to the reference value for São Paulo, the concentrations extracted by aqua regia were higher. Fadigas et al. (2002) observed similar values using the upper quartile (P75), with the Pb contents of the Guamium basin being similar to those found in this study. In Chinese soils, the Pb QRV was close to that adopted by the state of São Paulo (Jiang et al., 2025).

The Cr and Zn contents were similar to the quality reference values established by CETESB (2021) (Table 1). In the states of Mato Grosso and Rondônia (Santos & Alleoni, 2013), the QRV of Cr was higher when using the EPA 3051 and aqua regia methods, while the QRV of Zn was lower. Similar results were also found in soils from Espírito Santo (Paye et al., 2010). Conversely, the QRVs of Cr and Zn in soils from the Netherlands, Spain, and China were higher than those reported for the Brazilian states (Cancela et al., 2004; Jiang et al., 2025).

Both the P75 and P90 of Co were lower than the QRV established by CETESB (2021), and corresponded to approximately half the QRV adopted for the state of São Paulo (Table 1). These values are similar to those reported by Fadigas et al. (2002), who analyzed 162 samples of the main Brazilian soil classes, as well as those observed in Asian soils (Su & Yang, 2008). The reference value determined for the state of Espírito Santo is similar to that of São Paulo (Paye et al., 2010). However, the QRVs of Co for the soils of Mato Grosso and Rondônia (Santos & Alleoni, (2013) were higher than those of the other Brazilian states.

### Final considerations

Defining QRVs on a regional scale, such as that of micro–watersheds, allows for the precise capture of geological and land–use particularities that control the natural distribution of PTEs, thus avoiding underestimation and overestimation of environmental risks associated with the use of single values at state or national scales.

The concentrations of potentially toxic elements in the soils of the Guamium watershed were, in some cases, higher than the reference values for water quality established for the state of São Paulo. Although the samples were collected in areas under agricultural and urbanized use, the concentrations of PTEs, in general, as well as the QRVs estimated for the watershed, remained lower than the values reported in Brazil and other countries.

Arsenic and copper had concentrations higher than the QRVs defined by the state environmental agengy, which indicates the need for special attention to these elements in the environmental management of the basin. In addition, the adoption of the 90th percentile (P90) as a statistical criterion resulted in higher reference values for the Guamium basin, when compared to the QRVs adopted for the state of São Paulo, which reinforces the importance of the regional adequacy of these parameters.

This study contributes to advancing the understanding of the natural and anthropogenic variability of PTEs in humid tropical soils and provides technical support for the establishment of more representative reference values in highly industrialized regions. The results can support environmental monitoring actions, risk assessment, land use planning and decision-making by environmental agencies, as well as serve as a basis for future revisions of QRVs in areas with similar geological characteristics.

## Supplementary Information

Below is the link to the electronic supplementary material.

Supplementary file1 (DOCX 35 KB)

## Data Availability

No datasets were generated or analysed during the current study.
