# Supplementary material for: Concentration of potentially toxic elements in soils of an intensively agricultural and industrial watershed
Source: Environ Geochem Health. 2026 Aug 7;48(12):510. doi: 10.1007/s10653-026-03400-8 (PMC13451246; doi:10.1007/s10653-026-03400-8)
Supplement: Supplementary file 1 [file 10653_2026_3400_MOESM1_ESM.docx]

**SUPPLEMENTARY MATERIAL**

**Concentration of potentially toxic elements in soils of an intensively agricultural and industrial watershed**

Luís Reynaldo Ferracciú Alleoni, Evandro Barbosa da Silva, Adriele Laena Ferreira de Moraes

*University of São Paulo (USP), Luiz de Queiroz College of Agriculture (ESALQ), Piracicaba, São Paulo, Brazil, 13418900.*

**Table 1S.**

Descriptive analysis of the physical and chemical attributes of the most superficial soil layer (0–0.2 m). (n = 30).

| **Variable** | **Average** | **Minimum** | **Maximum** | **Median** | **Standard Deviation** |
| --- | --- | --- | --- | --- | --- |
| pH _(0.01M CaCl2)_ | 5.4 | 4.3 | 7.0 | 5.4 | 0.7 |
| **mmol_c_ kg^-1^** | | | | | |
| Ca | 22 | 5 | 65 | 21 | 13 |
| Mg | 12 | 1 | 38 | 11 | 8 |
| K | 3.1 | 0.1 | 9 | 3.1 | 2.2 |
| Al^+3^ | 2.1 | 0.0 | 15.3 | 0.4 | 4.1 |
| H + Al | 29 | 0.1 | 64 | 28 | 14 |
| CEC | 66 | 48.3 | 107 | 65 | 13 |
| **g kg^-1^** | | | | | |
| OC | 28 | 11 | 45 | 28 | 8 |
| Sand | 364 | 137 | 764 | 325 | 168 |
| Silt | 133 | 34 | 200 | 144 | 45 |
| Clay | 504 | 201 | 729 | 541 | 144 |
| Fe_2_O_3 DCB_ | 81 | 27 | 142 | 80 | 32 |
| Al_2_O_3 DCB_ | 16 | 9 | 24 | 17 | 4 |
| MnO _DCB_ | 0.6 | 0.2 | 1.2 | 0.5 | 0.3 |
| Fe_2_O_3 OX_ | 56 | 0.0 | 161 | 51 | 34 |
| Al_2_O_3 OX_ | 5 | 0.2 | 8 | 5 | 2 |
| MnO _OX_ | 1.5 | 0.0 | 2.2 | 1.5 | 0.4 |
| SiO_2 SH_ | 170 | 65 | 232 | 175 | 42 |
| Al_2_O_3 SA_ | 194 | 79 | 269 | 197 | 47 |
| Fe_2_O_3 SA_ | 432 | 17 | 1063 | 414 | 222 |
| MnO _SA_ | 0.6 | 0.2 | 1.2 | 0.5 | 0.3 |
|  |  |  |  |  |  |
| Ki | 0.9 | 1.5 | 2.7 | 1.5 | 0.4 |
| Kr | 0.2 | 0.7 | 1.1 | 0.7 | 0.2 |

CEC – cation exchange capacity, OC – organic carbon, SH – extracted with sodium hydroxide, SA – extracted with sulfuric acid, DCB – extracted with sodium dithionite-citrate-bicarbonate, OX – extracted with acid ammonium oxalate, LQ – limit of quantification (0.51 mg kg^-1^).

**Table 2S.**

Pearson correlation with significant values (*p* < 0.05) between contents of potentially toxic elements and soil attributes (n = 98)

|  | **Depth** | **pH** | **OC** | **CEC** | **Clay** | **Silt** | **Sand** | **As** | **Co** | **Cr** | **Cu** | **Ni** | **Pb** | **Zn** | **Fe_2_O_3_**  **_DCB_** | **Al_2_O_3_**  **_DCB_** | **MnO**  **_DCB_** | **Fe_2_O_3_**  **_OX_** | **Al_2_O_3_**  **_OX_** | **MnO**  **_OX_** |
| --- | --- | --- | --- | --- | --- | --- | --- | --- | --- | --- | --- | --- | --- | --- | --- | --- | --- | --- | --- | --- |
| **As** | -0.11 | -0.14 | 0.20 | 0.13 | **0.36** | 0.11 | **-0.36** | 1.00 |  |  |  |  |  |  |  |  |  |  |  |  |
| **Co** | -0.03 | **0.41** | -0.07 | -0.10 | 0.04 | **0.41** | -0.14 | -0.12 | 1.00 |  |  |  |  |  |  |  |  |  |  |  |
| **Cr** | 0.15 | -0.16 | -0.04 | 0.03 | -0.02 | 0.11 | 0.00 | 0.19 | **0.41** | 1.00 |  |  |  |  |  |  |  |  |  |  |
| **Cu** | 0.06 | **0.40** | -0.19 | **-0.30** | **0.35** | **0.29** | **-0.40** | -0.04 | **0.83** | **0.24** | 1.00 |  |  |  |  |  |  |  |  |  |
| **Ni** | 0.12 | 0.01 | -0.15 | -0.08 | -0.06 | **0.39** | -0.04 | 0.13 | **0.64** | **0.76** | **0.53** | 1.00 |  |  |  |  |  |  |  |  |
| **Pb** | -0.02 | 0.08 | 0.03 | 0.02 | **-0.26** | 0.13 | **0.20** | **-0.29** | **0.58** | **0.56** | **0.44** | **0.57** | 1.00 |  |  |  |  |  |  |  |
| **Zn** | **-0.20¹** | **0.54** | **0.27** | 0.14 | 0.20 | **0.49** | **-0.30** | 0.15 | **0.67** | 0.14 | **0.65** | **0.32** | **0.22** | 1.00 |  |  |  |  |  |  |
| **Fe_2_O_3DCB_** | 0.11 | **0.22** | **-0.25** | **-0.36** | **0.74** | 0.16 | **-0.72** | **0.26** | **0.38** | 0.08 | **0.70** | 0.15 | 0.03 | **0.38** | 1.00 |  |  |  |  |  |
| **Al_2_O_3DCB_** | 0.01 | -0.13 | -0.18 | -0.12 | **0.60** | **0.26** | **-0.62** | **0.40** | -0.10 | -0.15 | 0.08 | -0.02 | **-0.42** | -0.02 | **0.43** | 1.00 |  |  |  |  |
| **MnO_DCB_** | **-0.29** | **0.39** | **0.31** | 0.17 | 0.00 | **0.54** | -0.14 | 0.16 | **0.57** | 0.18 | **0.49** | **0.33** | **0.32** | **0.60** | **0.29** | -0.06 | 1.00 |  |  |  |
| **Fe_2_O_3OX_** | -0.03 | **0.48** | -0.14 | **-0.28** | **0.35** | **0.20** | **-0.38** | -0.07 | **0.73** | 0.12 | **0.81** | **0.26** | **0.37** | **0.50** | **0.70** | 0.15 | **0.39** | 1.00 |  |  |
| **Al_2_O_3OX_** | 0.13 | -0.09 | -0.02 | -0.10 | **0.45** | -0.10 | **-0.39** | **0.35** | **0.23** | 0.20 | **0.35** | 0.08 | 0.08 | **0.24** | **0.52** | **0.34** | -0.02 | **0.49** | 1.00 |  |
| **MnO_OX_** | **-0.31** | **0.50** | 0.18 | 0.06 | -0.07 | **0.31** | -0.01 | -0.10 | **0.30** | -0.02 | **0.25** | 0.08 | **0.26** | **0.42** | 0.08 | -0.15 | **0.53** | **0.32** | -0.01 | 1.00 |

OC – organic carbon, CEC – cation exchange capacity, DCB – extracted with sodium dithionite-citrate-bicarbonate, OX – extracted with acid ammonium oxalate.

**Table 3S.**

Distribution of the contents of potentially toxic elements in the Guamium watershed soils as a function of distance to Guamium River, and Pearson correlation with significant values (*p* < 0.05) between the levels of potentially toxic elements and the distance from the source (n=14).

| **Soil** | **Distance** | **As** | **Cd** | **Co** | **Cr** | **Cu** | **Ni** | | **Pb** | | **Zn** | |
| --- | --- | --- | --- | --- | --- | --- | --- | --- | --- | --- | --- | --- |
|  | km | -------------------------------------------------------------- mg kg^-1^ ------------------------------------------------------------------------------ | | | | | | | | | |  |
| LVA^1^-2 | 0 | **7.9 ± 0.7** | <LQ | 1.5 ± 0.2 | 32 ± 4.0 | 22 ± 1.6 | 3.7 ± 0.8 | | 15 ± 0,6 | | 24 ± 1.8 | |
| LVA-1 | 1.2 | **6.6 ± 1.1** | <LQ | 0.6 ± 0.2 | 17 ± 1.9 | 14 ± 2.2 | 1.2 ± 0.8 | | 17 ± 0,6 | | 15 ± 3.5 | |
| LV^1^-1 | 1.8 | **13 ± 2.2** | <LQ | 2.4 ± 0.2 | 35 ± 3.6 | 45 ± 4.4 | 4.9 ± 1.6 | | 13 ± 0,6 | | 32 ± 8.7 | |
| GX^2^-1 | 2.4 | **16 ± 1.6** | <LQ | 5.5 ± 1.1 | 29 ± 7.2 | 33 ± 6.2 | 9.6 ± 3.8 | | 2.7 ± 8.7 | | 56 ± 21 | |
| GX-2 | 2.7 | **8.6 ± 2.8** | <LQ | 7.1 ± 0.6 | 39 ± 22 | 42 ± 4.9 | 32 ± 31 | | 29 ± 3.5 | | 31 ± 4.4 | |
| LVA-3 | 3.8 | **5.4 ± 3.0** | <LQ | 4.5 ± 0.1 | 40 ± 2.6 | 12 ± 1.2 | 8.0 ± 2.2 | | 15 ± 0.8 | | 15 ± 2.3 | |
| LV-5 | 4.3 | **11 ± 3.7** | <LQ | 4.5 ± 3.2 | 50 ± 5.2 | 35 ± 26 | 19 ± 7.8 | | 31 ± 1.8 | | 22 ± 17 | |
| LV-3 | 5.0 | **15 ± 5.8** | <LQ | 3.0 ± 0.5 | 23 ± 1.5 | 30 ± 2.8 | 8.5 ± 3.2 | | 4.8 ± 1.2 | | 25 ± 5.1 | |
| LV-4 | 8.3 | **5.6 ± 2.5** | <LQ | 12 ± 1.3 | 34 ± 1.5 | 87 ± 5.1 | 26 ± 5.7 | 28 ± 1.7 | | 50 ± 7.7 | | |
| LV-2 | 10.7 | **8.5 ± 4.1** | <LQ | 1.9 ± 0.5 | 25 ± 0.8 | 30 ± 8.2 | 2.4 ± 3.0 | | 6.9 ± 0.6 | | 16 ± 6.9 | |
| **Pearson** | - | -0.57* | 0.14 | -0.23 | 0.03 | -0.14 | -0.04 | | 0.18 | | -0.57* | |

^1^Udic Oxisol; ^2^Aquic Entisol; LQ – limit of quantification.
